# Evidences of Early Senescence in Multiple Myeloma Bone Marrow Mesenchymal Stromal Cells

**DOI:** 10.1371/journal.pone.0059756

**Published:** 2013-03-21

**Authors:** Thibaud André, Nathalie Meuleman, Basile Stamatopoulos, Cécile De Bruyn, Karlien Pieters, Dominique Bron, Laurence Lagneaux

**Affiliations:** 1 Laboratory of Clinical Cell Therapy, Université Libre de Bruxelles (ULB), Institut Jules Bordet, Brussels, Belgium; 2 Hematology Department, Jules Bordet Institute, Brussels, Belgium; University of Sao Paulo - USP, Brazil

## Abstract

**Background:**

In multiple myeloma, bone marrow mesenchymal stromal cells support myeloma cell growth. Previous studies have suggested that direct and indirect interactions between malignant cells and bone marrow mesenchymal stromal cells result in constitutive abnormalities in the bone marrow mesenchymal stromal cells.

**Design and Methods:**

The aims of this study were to investigate the constitutive abnormalities in myeloma bone marrow mesenchymal stromal cells and to evaluate the impact of new treatments.

**Results:**

We demonstrated that myeloma bone marrow mesenchymal stromal cells have an increased expression of senescence-associated β-galactosidase, increased cell size, reduced proliferation capacity and characteristic expression of senescence-associated secretory profile members. We also observed a reduction in osteoblastogenic capacity and immunomodulatory activity and an increase in hematopoietic support capacity. Finally, we determined that current treatments were able to partially reduce some abnormalities in secreted factors, proliferation and osteoblastogenesis.

**Conclusions:**

We showed that myeloma bone marrow mesenchymal stromal cells have an early senescent profile with profound alterations in their characteristics. This senescent state most likely participates in disease progression and relapse by altering the tumor microenvironment.

## Introduction

Multiple myeloma (MM) is a malignant disorder of post-germinal center B-cells characterized by a monoclonal expansion of secreting plasma cells (PCs) in bone marrow (BM). MM is associated with a variety of well-known clinical manifestations, including skeletal destruction, renal failure, anemia, hypercalcaemia and recurrent infections [1]. MM represents approximately 1% of all malignant tumors, 10% of hematopoietic neoplasms and 2% of cancer deaths [2]–[4]. Despite recent advances in cancer therapy (e.g., Thalidomide, Lenalidomide and Bortezomib), MM remains an incurable disease with a median survival ranging from 29 to 62 months depending on the stage of disease [5].

MM is also characterized by a premyelomatous and asymptomatic stage termed monoclonal gammopathy of undetermined significance (MGUS). MGUS is the most frequent clonal plasma-cell disorder in the population, and it transforms into MM in 25–30% of patients [6]–[8]. The progression of myeloma from a benign precursor stage to the deadly malignancy depends on a complex set of factors that are not yet fully understood [9].

It is now well-established that BM constitutes a microenvironment required for differentiation, maintenance, expansion, and drug resistance development in MM cell clone [10]–[12]. The bone marrow microenvironment (BMME) is a complex network of heterogeneous cells which include osteoclasts, lymphoid cells, endothelial cells, mesenchymal stromal cells and their progeny (i.e., osteoblasts and adipocytes), as well as an extracellular and liquid compartment organized in a complex architecture of sub-microenvironments (or so-called ‘niches’) within the protective coat of mineralized bone. The BMME facilitates the survival, differentiation, and proliferation of hematopoietic cells through direct and indirect contacts.

In MM, the balance between the cellular, extracellular, and liquid compartments within the BM is profoundly disturbed. Indeed, bone marrow mesenchymal stromal cells (BM-MSCs) support MM cell growth by producing a high level of interleukin-6 (IL-6), a major MM cell growth factor [13]. BM-MSCs also support osteoclastogenesis and angiogenesis [14], [15]. Previous studies have suggested that the direct (via VLA-4, VCAM-1, CD44, VLA-5, LFA-1, and syndecan-1) and indirect (via soluble factors) interactions between MM plasma cells and BM-MSCs result in constitutive abnormalities in BM-MSCs. In particular, MM BM-MSCs express less CD106 and fibronectin and more DKK1, IL-1β, and TNF-α compared with normal BM-MSCs [16]–[18]. Furthermore, the clinical observation that bone lesions in MM patients do not heal even after response to therapy seems to support the idea of a permanent defect in MM BM-MSCs [19], [20].

The aims of this study were to investigate the constitutive differences between MM BM-MSCs and healthy donors’ (HD) BM-MSCs and to evaluate the impact of recent treatments (Thalidomide, Lenalidomide and Bortezomib) on MM BM-MSCs. We carried out microarray analyses of BM-MSCs derived from MM patients and healthy donors with an Affymetrix GeneChip covering the entire genome. In addition, we evaluated various MM BM-MSCs characteristics such as proliferation capacity, osteoblastogenesis, the cytokine and chemokine expression profile, hematopoietic support, and immunomodulatory activity.

## Design and Methods

### Patients

Each sample was obtained after receiving written informed consent from patients and donor volunteers and after approval from the Jules Bordet Ethical Committee. Fifty-seven patients with multiple myeloma or MGUS were included in this study and their characteristics are listed in Table S1. Each treated MM patients were under remission at the moment of harvesting and did not receive a graft. Twenty BM samples were obtained from healthy donors with a mean age of 54 years (ranging from 44 to 69) and a sex ratio of 12/8 (M/F).

### Isolation, Culture and Characterization of BM-MSCs

Bone marrow was harvested from the sternum or iliac crest of patients. BM-MSCs were isolated by the classical adhesion method and cultivated as previously described [21]. The harvested cells were analyzed by flow cytometry. Briefly, the cells were washed with phosphate buffered saline (PBS; GmbH, Bergisch, Germany) and incubated for 20 min with propidium iodide (PI) and the following monoclonal antibodies (data not shown): CD105-FITC (Ancell Corporation, Bayport, MN, USA), CD73-PE (BD Biosciences Pharmingen, Erembodegen, Belgium), CD146-PC5 (Beckman Coulter, Marseille, France), CD31-FITC (Miltenyi Biotec, Leiden, The Netherlands), CD45-PC7 (BD Biosciences), CD166-PE (BD Biosciences), CD38-FITC (Miltenyi Biotec), CD19-PC5 (Beckman Coulter) and CD138-PE (BD Biosciences). After washing with MACSQuant Running Buffer (Miltenyi Biotec), the cells were fixed with 4% formaldehyde solution. Data were acquired using MACSQuant Analyzer (Miltenyi Biotec) and analyzed using FCS Express 4 Flow Cytometry software (De Novo Software, Los Angeles, CA, USA).

### Cell Proliferation

To estimate the number of mesenchymal clonogenic cells, a colony-forming unit fibroblast (CFU-F) assay was performed after each passage. Briefly, after TrypLE Select detachment, 5000 cells were plated per 10 cm^2^ Petri dish containing culture medium at 37°C in 5% CO_2_. After 10 days, the medium was discarded, and the adherent cells were stained with May-Grunwald Giemsa as previously described [22]. Fibroblastic colonies of >50 cells were scored using an inverted microscope. To evaluate the total number of CFU-F after expansion (passage 1), we calculated the cumulative number of cells obtained and reported the number of CFU-F obtained for 5000 cells to the cumulative number of cells obtained after expansion.

### SA β-Galactosidase Staining

Ten thousand BM-MSCs cells were plated in a 48 well-plate for 24 h before staining cells with the Senescence Detection Kit (BioVision, Milpitas, CA, USA). Number of blue cells out 100 total cells was scored using an inverted microscope. The results were presented as the mean percentage (± SEM) of blue cells compared to total counted cells.

### Microarrays

Three healthy donors (48, 50, 58 years) and four untreated myeloma patients (47, 52, 56, 65 years) were included in the analyses. Total RNA from each cell culture was extracted in a single step using TriPure Isolation Reagent (Roche Applied Science, Vilvoorde, Belgium). Microarray analysis was performed with 1.5 µg RNA using Affymetrix GeneChip® Human Genome U133 Plus 2.0 Arrays, which contained more than 54,000 probe sets for analysis of approximately 47,000 transcripts (Affymetrix, High Wycombe, UK). Amplification, hybridization, and scanning were performed according to standard Affymetrix protocols (www.affymetrix.com). The data discussed in this publication have been deposited in NCBI’s Gene Expression Omnibus and are accessible through GEO Series accession number GSE36474 (http://www.ncbi.nlm.nih.gov/geo/query/acc.cgi?token=ltazpowigoiekji&acc=GSE36474).

### Bioinformatic Analysis

A comparative gene expression profile was determined for 7 subjects (3 healthy donors and 4 untreated MM patients). We identified significant differences between sample groups using BRB ArrayTools (Biometric Research Branch, National Cancer Institute, Bethesda, MA, USA). Only genes defined as present by the Affymetrix algorithm in at least 30% of either of the two groups were considered for further analysis (26,553 ESTs). We performed two-sample t tests (with a random variance model) on the two groups for each gene. We then addressed the problem of multiple comparison by estimating the false discovery rate (FDR) as the ratio of the expected number of false positives at a given p value threshold to the number of positives actually found.

### Quantitative Real-Time PCR (qRT-PCR)

Total RNA extraction from each cell culture was performed as described above. We performed the reverse transcription reaction with 1 µg RNA using qScript cDNA SuperMix (Quanta Biosciences). Transcripts were quantified by qRT-PCR using 20 ng of cDNA, SYBR Green PCR Master Mix (Applied Biosystems, Lennik, Belgium) and 0.32 µM forward and reverse primers. The primers were designed with Primer Express 2.0 software (Applied Biosystems) or ProbeFinder online software (Roche) and are available in Table S2. To control variations in input RNA amounts, the GAPDH gene was used as a housekeeping gene to quantify and normalize the results. The reactions were carried out using the ABI Prism 7900 HT system (Applied Biosystems). In all cases, dissociation curves were generated and the specificity of the PCR reactions was confirmed. The comparative ΔΔCt method was used for the data analysis. To evaluate the fold change compared to HD, data were normalized with the GAPDH genes to obtain the ΔCt and were after calibrated with the geometric mean of the HD ΔCt to generate the ΔΔCt. Fold changes were then calculated as fold change = 2^−ΔΔCt^.”

### Cell Differentiation

We analyse the osteogenic differentiation of BM-MSCs as previously described [23]. Briefly, 10^4^ BM-MSCs cells were plated in a 24-well plate for 24 h before replacing the culture medium with 250 µL NH OsteoDiff Medium (Miltenyi Biotec). Cells were fed every week by changing the medium. At day 7, 14 and 21, calcium mineralization and ALP activity were assessed. The calcium deposits were harvested by a HCl solution and the concentration was determined by colorimetry as described by the manufacturer (QuantiChrom Calcium Assays Kit; BioAssay Systems, Hayward, CA, USA). For the alkaline phosphatase (ALP) activity assay, cells were recovered by TrypLE Select detachment, centrifuged at 13,000 × *g* and resuspended in 100 µL of 150 mM NaCl, 20 mM TrisHCl and 0.1% Triton X-100. Cells were frozen and thawed 3 times before measuring the ALP activity by LabAssay ALP (Wako Chemicals, Richmond, VA, USA) following the manufacturer’s protocol. In these samples, we also determined the protein concentration by Bradford protein assay (Bio-Rad, Hercules, CA, USA).

### Cytokine Expression

Briefly, 10^5^ BM-MSCs were plated in a 24-well plate for 24 h before replacing the culture medium with DMEM supplemented with 2 mM L-glutamine, 50 U/ml of penicillin, and 50 µg/ml of streptomycin. Cells were incubated for 48 h and then the conditioned media were harvested and stored at –20°C until use. The RayBio Human Cytokine Antibody Array (Tebu-Bio, Boechout, Belgium) was used to evaluate cytokine levels in conditioned media. We selected 30 different cytokines: BDNF, EGF, G-CSF, GM-CSF, HGF, IFNγ, IGF-I, IGF-II, IL-10, IL-1β, IL-6, IL-7, IL-8, MCP-1, MCP-3, MIP-1α, MIP-1β, MMP-2, MMP-9, OPG, PDGF-bb, RANTES, SCF, SDF-1, TGF-β, TIMP-1, TIMP-2, TIMP-4, TNF-α, and VEGF. Chemiluminescence was measured using a LAS-3000 imaging system (FujiFILM Europe, Belgium) with exposure in 10 sec intervals and analyzed using an Advanced Image Data Analyzer (AIDA). A positive control was used to normalize and quantify the results. The cytokine concentration in the culture media was determined by enzyme-linked immunosorbent assay (ELISA) for IL-6, DKK1, VEGF, GDF-15 and TGF-ß according to the manufacturer’s instructions (Quantikine, R&D Systems, Abingdon, UK).

### Hematopoietic Support

#### Blast-Colony Forming Cell (Bl-CFC) assay

This assay evaluates the ability of MSCs to sustain the survival and proliferation of early hematopoietic progenitor cells, which has been previously described (23). Briefly, 5×10^4^ MSCs, obtained between P1 and P3, were plated in DMEM/FBS in 4-well plates and grown until confluent. A total of 5×10^3^ CD34^+^ cells purified from umbilical cord blood (UCB) using MidiMACS separation (Miltenyi Biotec) were added to the feeder-layer, and 250 µL semisolid culture medium without cytokine (Stemcell Technologies, Grenoble, France) was added onto the cells. Refringent colonies of >20 cells, closely attached to the feeder layer, were counted using an inverted microscope after 7 days at 37°C. *Long-Term Culture-Initiating Cell (LTC-IC) assay*: Dexter-type long-term culture allows for the evaluation of primitive hematopoietic cells [24]. Fifty thousand MSCs, obtained between P1 and P3, were plated in DMEM/FBS in 4-well plates and grown until confluent. A total of 2.5×10^4^ CD34^+^ cells, in 500 µL DMEM/FBS, were added to the feeder-layer, and every week, half of the medium was replaced. After five weeks, adherent and non-adherent cells were harvested and counted. Twenty microliters of the harvested cells were replated in a 4-well plate with 250 µL semisolid MethoCult H4536 with EPO (Stemcell Technologies). Cultures were kept at 37°C for 7 days, and then colonies (CFU-GM and BFU-e) of >20 cells were counted using an inverted microscope.

### Allogeneic Mixed Leukocyte reaction (MLR)

Approximately 1 or 3×10^5^ CD3^+^ T cells were co-cultured with 2, 10 or 20×10^4^ irradiated allogeneic peripheral blood mononuclear cells (PBMCs) in a 250 µl final volume in 96-well plates. Triplicates of these MLRs were prepared in the presence or absence of 10^4^ irradiated MSCs. After 4 days, 100 µl fresh medium containing Bromodeoxyuridine (BrdU; 50 µM final concentration) was added to each well for 24 hours. T cell proliferation was assessed by measuring BrdU incorporation using a colorimetric assay following manufacturer’s procedure (Roche Diagnostics, Mannheim, Germany). T cells, PBMCs, or MSCs cultured alone were used as controls for background determination. T cell proliferation values in MLRs performed without MSCs were considered as 100%.

### Statistical Analysis

Normal distribution of results was not assumed. The Kruskal-Wallis test and the Dunn’s multiple comparison test were used to analyze differences between groups. All tests were two sided. The level for significance was set at *p*<0.05 and the results were expressed as the means ± SEM. *p<0.05; **p<0.01; ***p<0.001. All analyses were performed using GraphPad Prism 5.

## Results

### MM BM-MSC Gene Expression Profile was Similar to that of Senescent BM-MSCs

**Figure 1:**
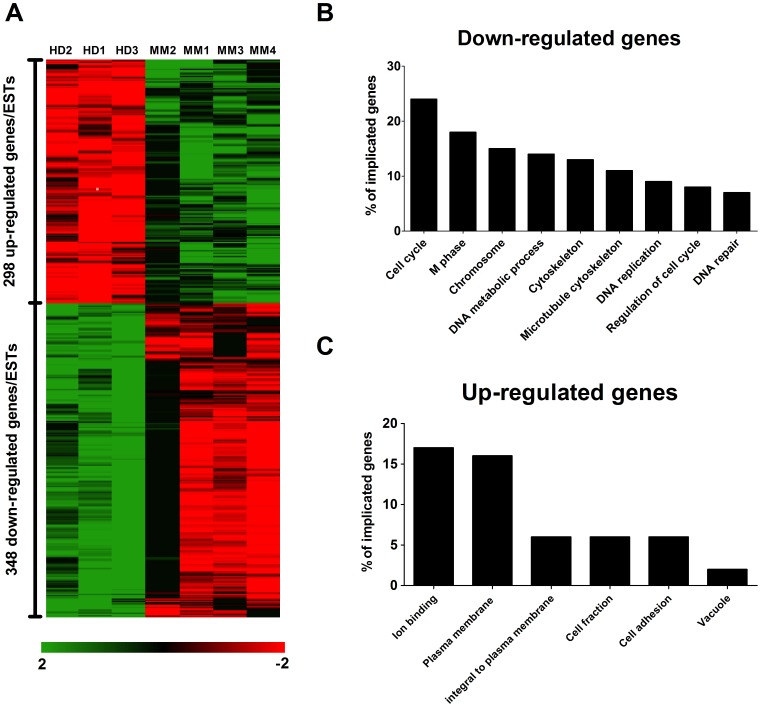
RNA expression profile in MM BM-MSCs is highly similar to senescent BM-MSCs. (**A**) Differential gene expression in 3 HD BM MSC samples and 4 untreated MM BM MSC samples was analyzed by Affymetrix GeneChip technology. In all, 419 ESTs were more than 2-fold up-regulated (green), and 537 were more than 2-fold down-regulated (red). Analysis demonstrated high changes in the global gene expression pattern in MM BM-MSCs. (**B and C**) Gene Ontology analysis (DAVID) was performed for the subsets of genes that were 2-fold up-regulated or 2-fold down-regulated, and the profile obtained was highly similar to the one observed by Wagner et al. in senescent BM-MSCs. The percentages of genes that contributed to representative categories are depicted (*p*<0.0001).

To obtain a global view of the differences between HD and MM patients, the genetic profiles of BM-MSCs from 3 HD and 4 untreated MM patients was determined. A fold change cutoff of 2-fold (increase or decrease) and *p*<0.001 allowed us to identify 646 probe sets (3.6%) that are differentially expressed with a false discovery rate <10%. Among them, 298 (43.9%) were up-regulated and 348 (56.1%) were down-regulated in MM BM-MSCs (Figure 1A). A functional annotation analysis using the DAVID Functional Annotation Tool [25], [26] demonstrated that the up-regulated genes (Figure 1B) in MM BM-MSCs were highly over-represented (*p*<0.0001) in the categories of ion binding (GO:43167), plasma membrane (GO:5886), integral to plasma membrane (GO:16021), cell fraction (GO:267), cell adhesion (GO:7155), and vacuole (GO:5773), whereas the down-regulated genes (Figure 1C) in MM BM-MSCs were highly over-represented in the categories of cell cycle (GO:7049), M phase (GO:279), chromosome (GO:5694), DNA metabolism (GO:6259), cytoskeleton (GO:5856), microtubule cytoskeleton (GO:15630), DNA replication (GO:6260), cell cycle regulation (GO:51726) and DNA repair (GO:6281). These profiles of biological functions, in which the majority of the genes/ESTs were involved, were very similar to those observed by Wagner et al. in the replicative senescence of human BM-MSCs [27]. Next, we focused our analysis on two biological processes, namely cell cycle and osteoblastogenesis. Many activators of the cell cycle such as cyclins A, B, E, D, H; CDKs 1, 2, 4, 6; or CDC25A, B and C were down-regulated. In addition, we observed a down-regulation of osteoblastogenesis activators (e.g., OPG, Smurf2, XCRR5, FosB, Hoxa10, Ets1) and an up-regulation of inhibitors or antagonists (e.g., Stat1/3, sFRP4, Hoxa2, Twist2, TLE1, EBF1, TGF-β2, SOX9). These data were validated by real-time RT-PCR for Ankyrin 3, CDC25A, Cyclin E, CDK2, FosB, Stat1, ANGPTL1 and 4 (Figure S1). We observed an overexpression of Sox9 mRNA but the difference between HD BM-MSCs and untreated MM BM-MSCs was not significant.

### MM BM-MSC Proliferation Capacity was Reduced

**Figure 2:**
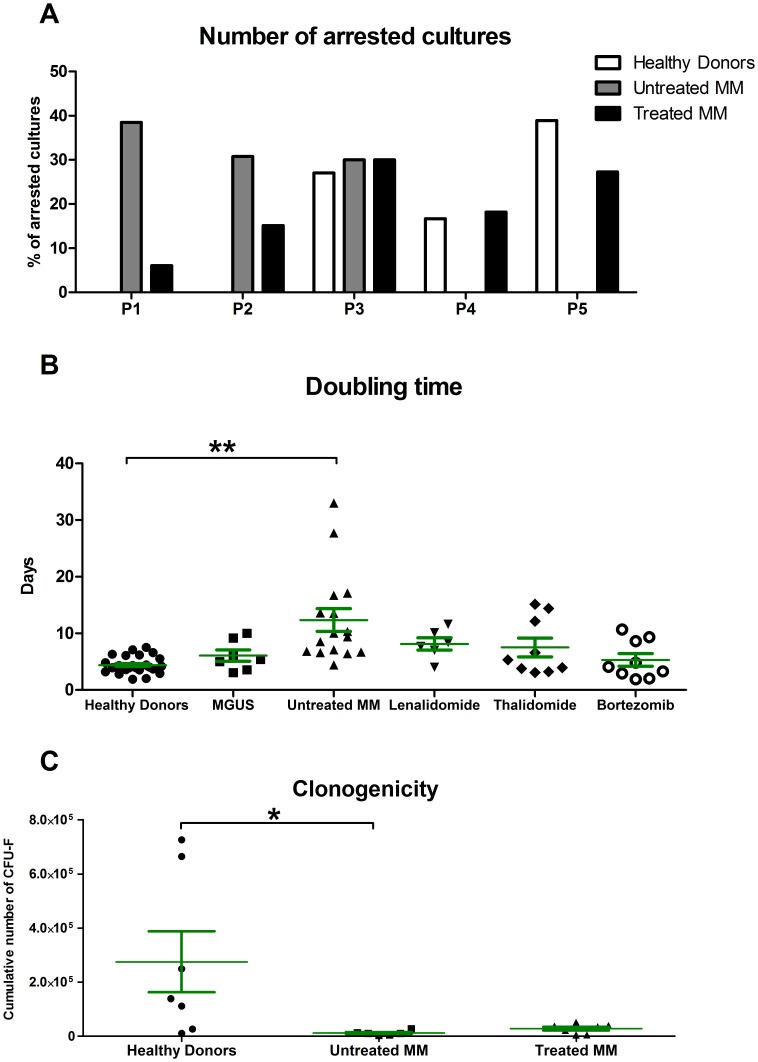
Reduced proliferation capacity of MM BM-MSCs. (**A**) Number of BM-MSC cultures in a growth arrested state at each passage. BM-MSCs from untreated MM patients stop at the first to third passage, whereas the BM-MSCs from healthy donors never stop before the third passage and could last until the seventh passage. BM-MSCs from treated MM patients have a mixed profile. (**B**) Doubling time of BM-MSC cultures from healthy donors (n = 17), MGUS patients (n = 5), untreated MM patients (n = 10) and patients treated with Lenalidomide (n = 5), Thalidomide (n = 7) or Bortezomib (n = 8). (**C**) Cumulative number of CFU-F at P1 for HD BM-MSCs (n = 6), untreated MM BM-MSCs (n = 5) and treated MM BM-MSCs (n = 6). Green bars represent the mean ± SEM. *p<0.05 and **p<0.01 compared to HD BM-MSCs.

Given the consistent alterations in the expression of cell cycle activators, we decided to evaluate the proliferative capacity of MM BM-MSCs. First, we observed that under routine *in vitro* culture conditions, a significant proportion of MM BM-MSC cultures failed (34.8%, 23/66 cases) in contrast with the normal counterpart (<5%, 1/21 cases). A majority of these failed MM BM-MSC cultures came from untreated MM patients (60.9%, 14/23 cases). Furthermore, we observed that BM-MSC cultures from untreated MM patients stop growing earlier (P1 to P3) than BM-MSC cultures from healthy donors (P3 to P5) and that BM-MSC cultures from treated MM patients have a mixed profile (P1 to P5 - Figure 2A). Second, we calculated the doubling time of BM-MSC cultures between the first and third passages (Figure 2B). The BM-MSCs from untreated MM patients took approximately 2-fold more time to double compared to BM-MSCs from healthy donors (p<0.01). Treatments tended to rescue the doubling time to normal levels, especially Bortezomib-based treatments. The doubling time of BM-MSCs from MGUS patients was not different compared to HD BM-MSCs. Finally, we evaluated the BM-MSC clonogenicity by calculating the cumulative number of Colony-Forming Unit Fibroblast (CFU-F – Figure 2C). We observed 23-fold more CFU-F at passage 1 with BM-MSCs from healthy donors than BM-MSCs from untreated MM patients (p<0.05). BM-MSCs from treated MM patients showed a decrease in cumulative CFU-F number, but the difference is not significant.

### MM BM-MSCs More Rapidly Entered Senescence

**Figure 3:**
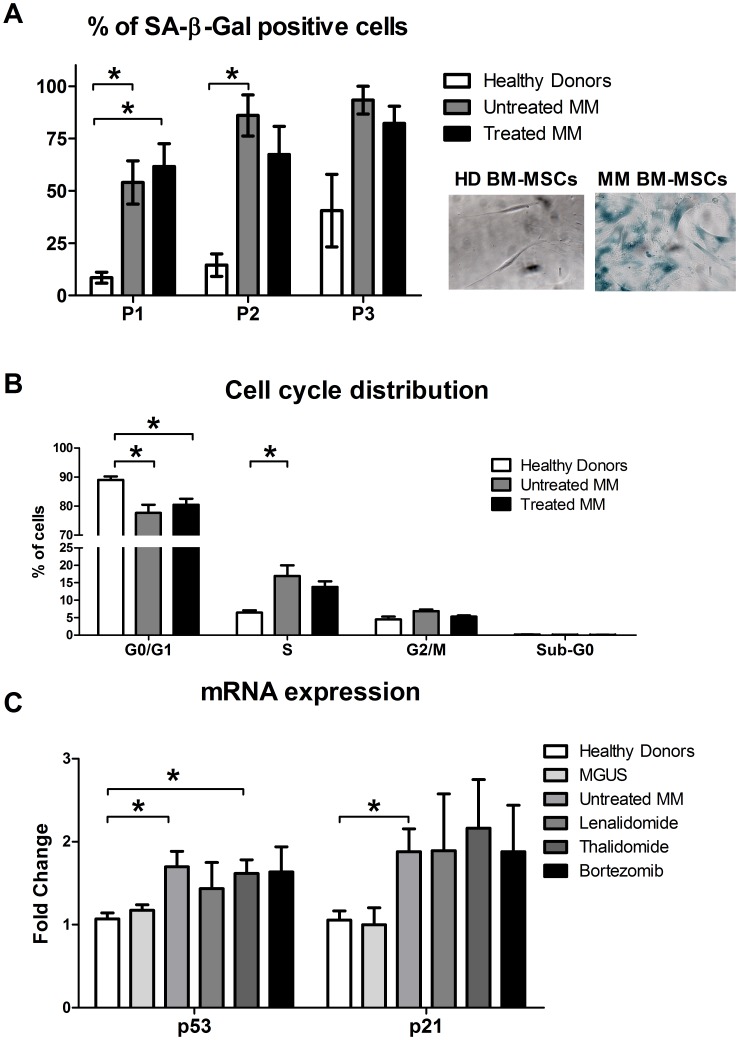
Early cellular senescence in MM BM-MSCs. (A - left) BM-MSCs were stained for senescence-associated β-Galactosidase (SA β-Gal) between passage 1 and 3. The mean percentage of SA β-Gal positive cells is higher in untreated (n = 9) and treated (n = 12) MM BM-MSCs compared to HD BM-MSCs (n = 6). (**A – right**) Representation of the SA β-Gal staining in HD BM-MSCs and untreated MM BM-MSCs. (**B**) Cell cycle distribution of BM-MSCs was determined by flow cytometric analysis after propidium iodide DNA staining. The graph indicates the percentages of cells in G_0_–G_1_, G_2_-M, S and Sub-G_0_ phases of the cell cycle. We observed a reduction in G_0_–G_1_ phase and an increase in S phase for BM-MSCs of untreated (n = 7) and treated (n = 12) MM patients compared to HD BM-MSCs (n = 9). (**C**) mRNA expression of p53 and p21 by MGUS patients (n = 4), untreated MM BM-MSCs (n = 6) and MM BM-MSCs treated by Lenalidomide (n = 6), Thalidomide (n = 8) and Bortezomib (n = 6), compared to HD BM-MSCs (n = 12). *p<0.05 compared to HD BM-MSCs.

In addition to the reduced growth rate, MM BM-MSCs became senescent as demonstrated by the expression of senescence-associated β-galactosidase between passages 1 and 3 (SA β-Gal; Figure 3A). It is known that senescence is associated with alterations in cell morphology with cells becoming much larger and having an irregular and flat shape. By flow cytometry, we measured the MM BM-MSC cell size compared to standard microbeads (Miltenyi Biotec) and observed an increase in the size of the MM BM-MSCs (32.55 µm ±0.19; n = 10) compared to the HD BM-MSCs (31.32 µm ±0.17; n = 15; p<0.001; data not shown). No difference was observed between MGUS and HD BM-MSCs. To investigate whether the decreased cell growth of MM BM-MSCs was due to decreased proliferation or increased apoptosis, the distribution of the cells in each cell cycle phase was compared (Figure 3B). No Sub-G_0_ peak, corresponding to hypodiploid apoptotic cells, was observed in our BM-MSC cultures (<1%). However, the percentage of untreated MM BM-MSCs in G_0_/G_1_ phase fell to 77.7±2.8% (vs 89±1.18%; p<0.05) whereas the percentage in S phase climbed to 16.96±3.05% (vs 6.44±0.64%; p<0.05) when compared to HD BM-MSCs. Finally, we evaluated the expression of cell cycle regulators using quantitative Real-Time PCR (qRT-PCR). We observed an up-regulation of p53 and p21 in untreated MM BM-MSCs compared to HD BM-MSCs (Figure 3C; p<0.05). In contrast, no difference was observed for p16 and pRB (data not shown) between HD BM-MSCs and MM BM-MSCs. In these experiments, no difference was observed between MM BM-MSCs from treated and untreated MM patients.

### Osteoblastogenesis was Altered in MM BM-MSCs

**Figure 4:**
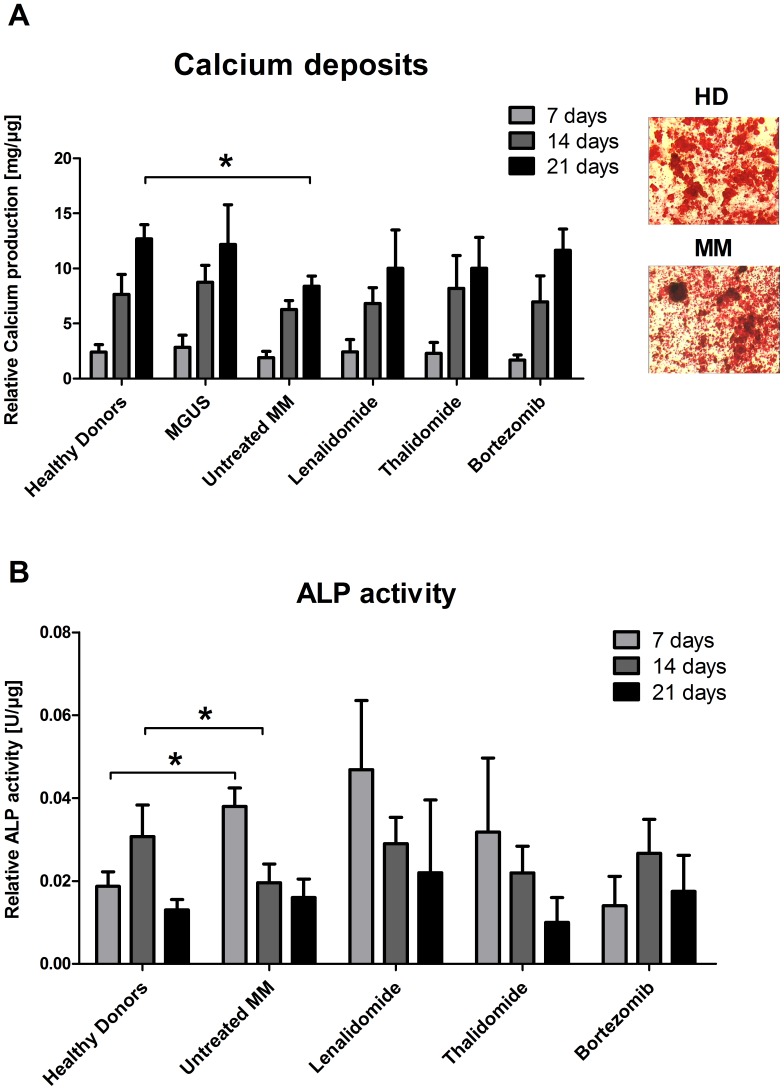
Reduced osteoblastogenesis in MM BM-MSCs. (A - left) Relative calcium production (mg of calcium/µg of total proteins) by HD BM-MSCs (n = 6) as well as BM-MSCs from MGUS patients (n = 3), untreated (n = 12) and MM patients treated by Lenalidomide (n = 5), Thalidomide (n = 6) and Bortezomib (n = 8) after 7, 14 and 21 days of differentiation into osteoblasts. (A – right) Alizarin Red staining of HD BM-MSCs and BM-MSCs from untreated MM patients after 21 days of differentiation. (B) Relative alkaline phosphatase (ALP - Units/µg of total proteins) activity in HD BM-MSCs (n = 9) as well as BM-MSCs from untreated (n = 11) and MM patients treated by Lenalidomide (n = 5), Thalidomide (n = 6) and Bortezomib (n = 5) after 7, 14 and 21 days of differentiation into osteoblasts. *p<0.05 compared to HD BM-MSCs.

Considering that abnormal differentiation is associated with senescence [28] and that MM is characterized by unbalanced bone turnover, we evaluated the osteoblastic differentiation potential of MM BM-MSCs by measuring the relative calcium production, the relative alkaline phosphatase (ALP) activity and mRNA expression during *in vitro* osteoblastogenesis. We observed that the relative production of calcium (Figure 4A) by MM BM-MSCs from untreated patients was reduced after 21 days of differentiation (p<0.05). The treatments tended to improve calcium production, and the MGUS BM-MSCs showed no difference when compared with HD BM-MSCs (preliminary results). We also observed that ALP (Figure 4B) is more active at the first step of the differentiation (after 7 days; p<0.05) in untreated MM BM-MSCs compared to HD BM-MSCs. By contrast, ALP is less active after 14 days of differentiation in untreated MM BM-MSCs (p<0.05).

### MM BM-MSCs had an Aberrant Secretory Profile

**Figure 5:**
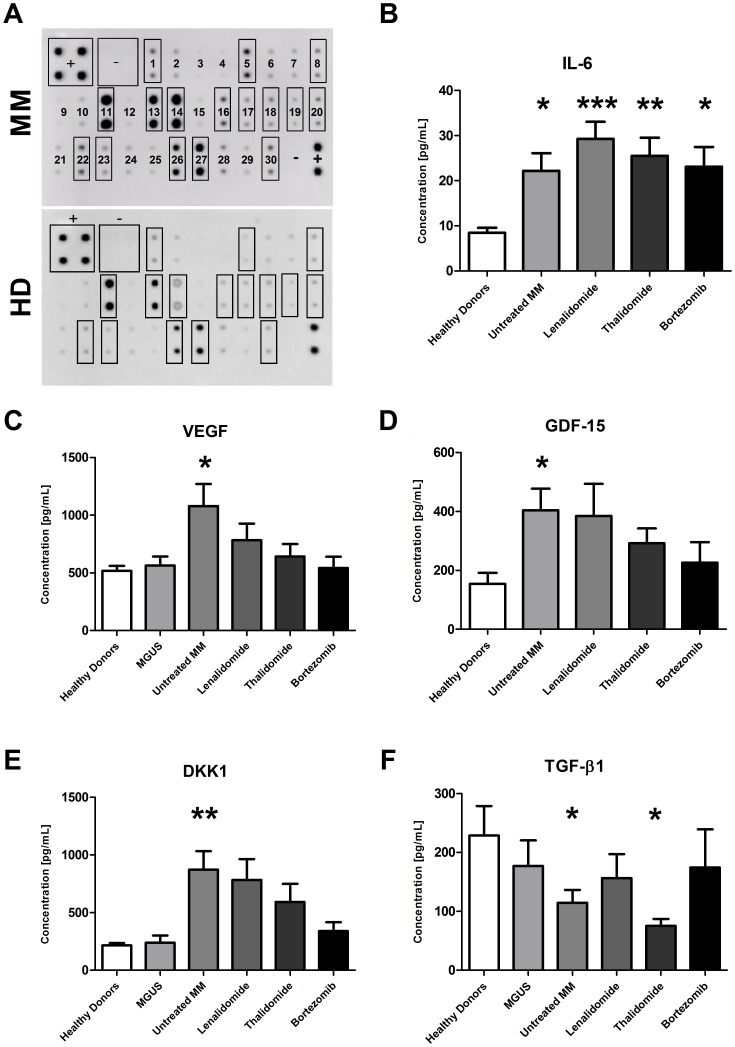
BM-MSC cytokine and chemokine expression profile. (A) Representative cytokine/chemokine antibody array associated with conditioned media (CM) from myeloma and healthy BM-MSCs. (+) and (−) represent the internal positive and negative controls and numbers represents the targeted cytokine: 1. BDNF/2. EGF/3. GCSF/4. GMCSF/5. HGF/6. IFN-γ/7. IGF-I/8. IGF-II/9. IL-10/10. IL-1β/11. IL-6/12. IL-7/13. IL-8/14. MCP-1/15. MCP-3/16. MIP-1α/17. MIP-1β/18. MMP-2/19. MMP-9/20. OPG/21. PDGF-BB/22. RANTES/23. SCF/24. SDF-1/25. TGF-β2/26. TIMP-1/27. TIMP-2/28. TIMP-4/29. TNF-α/30. VEGF. We observed an upregulation in MM BM-MSC CM of BDNF, HGF, IGF-II, IL-6, IL-8, MCP-1, MIP-1a, MIP-1b MMP2, MMP9, OPG, RANTES, SCF, TIMP-1, TIMP-2, and VEGF. (B, C, E and F) Levels of IL-6, VEGF, GDF-15, DKK1 and TGF-β1, measured by ELISA, in conditioned media obtained from healthy donors (n = 14), MGUS patients (n = 5), untreated MM (n = 11) and MM patients treated by Lenalidomide (n = 9), Thalidomide (n = 12) or Bortezomib (n = 8). *p<0.05; **p<0.01; ***p<0.001 compared to HD BM-MSCs.

MSC-conditioned media were obtained after 48 hours of culture in serum-free conditions and the levels of 30 cytokines/chemokines were measured by cytokine array. A typical secretion profile of BM-MSCs from untreated MM patients and healthy donors using a cytokine array membrane is shown in Figure 5A. Antibody array analysis from MM BM-MSCs revealed higher secretion of BDNF, HGF, IGF-II, IL-6, IL-8, MCP-1, MIP-1a, MIP-1b, MMP-2, MMP-9, OPG, RANTES, TIMP-1, TIMP-2, and VEGF in comparison to HD BM-MSCs (n = 4). By ELISA, we further analyzed five factors to confirm their increased production and to evaluate the impact of MM treatments on their secretion (Figure 5B). We observed an increase in IL-6, DKK1, VEGF, and GDF-15 production by MM BM-MSCs compared to HD BM-MSCs. The production of IL-6 was not influenced by any treatments whereas the one of VEGF was reduced by all treatments, the one of GDF-15 and DKK1 was reduced by Bortezomib-based and Thalidomide-based treatments in MM BM-MSCs compared to untreated MM BM-MSCs. Finally, we observed a decrease in TGF-β production by untreated MM BM-MSCs. Thalidomide-based treatments reduced TGF-β production whereas Lenalidomide and Bortezomib-based treatments increased TGF-β production when compared to untreated MM BM-MSCs.

### MM BM-MSCs More Efficiently Supported the Growth of CD34^+^ Cells

**Figure 6:**
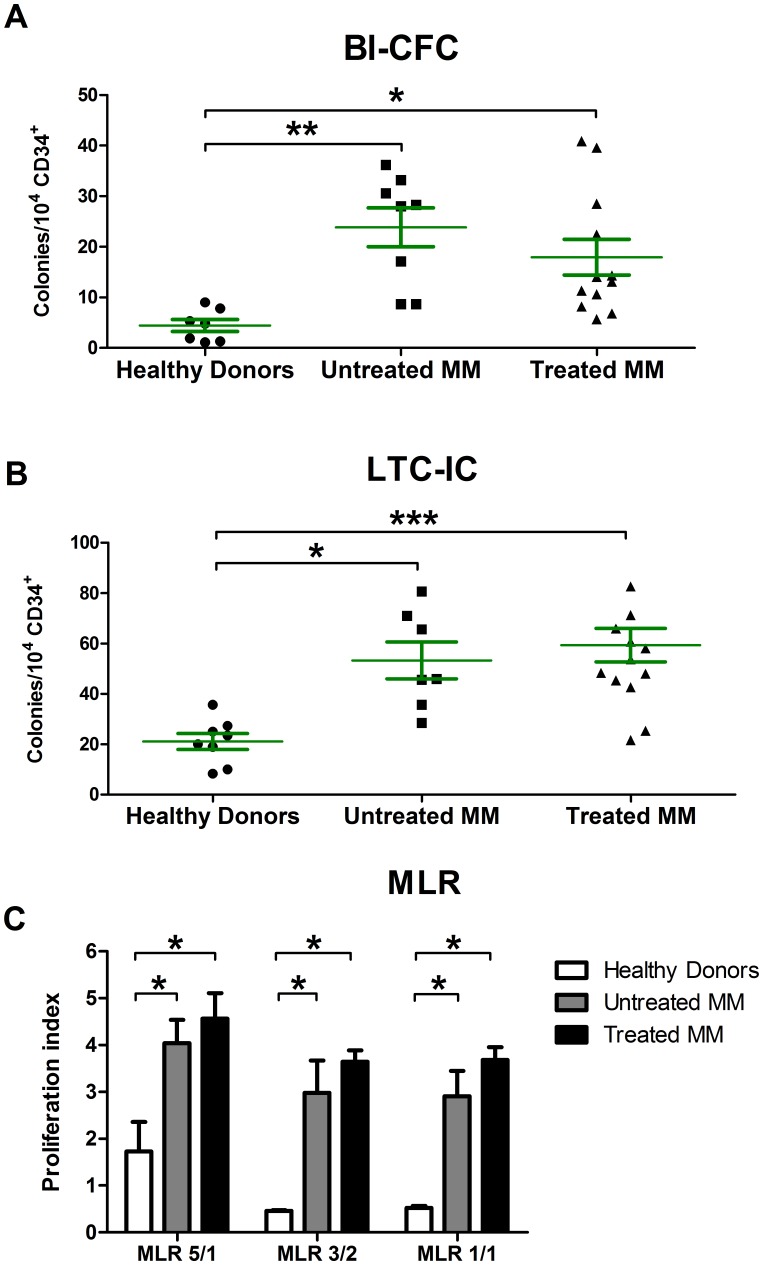
Altered MM BM-MSC hematopoietic support and immunomodulatory capacity. (A) Relative number of colonies per 10^4^ cord blood CD34^+^ cells cultured on BM-MSC confluent layers for 7 days. BM-MSCs from untreated (n = 6; Kruskal-Wallis test, statistic = 12.88; *P<0.05; Dunn’s multiple comparison test Δ rank sum = 14.00) and treated (n = 10) MM patients support the growth of CD34^+^ cells more efficiently compared to HD BM-MSCs (n = 6). (**B**) Number of secondary colonies formed by CD34^+^ cells cultured for 7 days in semi-solid medium with cytokines and erythropoietin, after 5 weeks of co-culture with HD or MM BM-MSCs. We observed an increased number of colonies in untreated (n = 7) and treated (n = 14) MM patients compared to HD (n = 8). Green bars represent the mean ± SEM. (**C**) Different CD3^+^/PBMC ratios were used to perform MLR in the presence of HD BM-MSCs (n = 5), untreated MM BM-MSCs (n = 5) and treated MM BM-MSCs (n = 6). T cell proliferation was assessed by BrdU incorporation assay after 5 days of co-culture. For each ratio, MM BM-MSCs had decreased inhibition capacity. *p<0.05 and **p<0.01 compared to HD BM-MSCs.

We evaluated the capacities of MM BM-MSC confluent layers to sustain the proliferation of cord blood CD34^+^ cells. For this purpose, we evaluated the Bl-CFC number after 7 days of co-culture in semi-solid medium without cytokine (Figure 6A). A mean of 24±4 Bl-CFC per 10^4^ CD34^+^ cells was observed for untreated MM BM-MSCs (n = 6; p<0.01), 18±3 for treated MM BM-MSCs (n = 10; p<0.05) and 5±1 for HD BM-MSCs (n = 6). We also evaluated the hematopoietic support capacities of MSCs in long-term culture conditions (Figure 6B). We observed that the production of secondary colony-forming cells was higher after co-culture with untreated MM BM-MSCs (53±7; n = 6; p<0.05) and treated MM BM-MSCs (59±7; n = 7; p<0.001) than with HD BM-MSCs (21±3; n = 7).

### The Inhibition of Allogenically Stimulated T Cells by MM BM-MSCs was Reduced

By MLR, we evaluated the growth inhibition of activated CD3^+^ cells, isolated from healthy donor peripheral blood, by MM and HD BM-MSCs. We observed an inhibition of CD3^+^ proliferation when they were co-cultured with HD BM-MSCs in CD3^+^/PBMC ratio of 1/1 and 3/2 (0.52 and 0.45, respectively). As previously described (21), the inhibition of proliferation was overcome when the CD3^+^ cells were too numerous in the co-culture (ratio 5/1∶1.72). Interestingly, at any ratio, we observed increased proliferation when CD3^+^ cells were co-cultured with MM BM-MSCs (3.40 to 5.23; p<0.05), which demonstrates their reduced immunomodulatory capacities (Figure 6C).

## Discussion

Multiple myeloma, as with other cancers, is characterized by a complex tumor microenvironment that confers support (e.g., growth factors, vascularization) and protection (e.g., drug resistance) to malignant cells [11], [12], [29]. Among the factors in the MM tumor microenvironment, BM-MSCs play a crucial role through their direct and indirect contacts with MM-PCs. One of the consequences of these contacts is the intrinsic abnormalities observed in MM BM-MSCs such as an overexpression of IL-6 and DKK1, a reduced inhibition of T lymphocyte proliferation and a distinct genomic profile [16]–[18], [30]. In this study, we analyzed various MM BM-MSC characteristics with the aim to better understand these abnormalities, their origins and their implications in pathophysiology.

We investigated the genetic profile of MM BM-MSCs by microarray. Compared to previous microarray analyses [16], [31], we observed more genes/ESTs differentially expressed (646 vs 183 and 79). Despite a partial overlap between these analyses and ours (e.g., ANGPTL4, GDF15, SERPINE1, CXCL12, SOX9, HOXB6, ANK3), the majority of the genes/ESTs observed by the two other studies were not present in ours (approximately 28 and 12% correlation) and no correlation were found between the two other analyses. These differences are mainly due to divergences in the protocol such as MSC harvesting method (BM aspiration vs bone biopsies), type of Affymetrix Genechip (U133 Plus2.0 with 54.000 probe sets vs U133A with 22.000 probe sets), number of samples (4 vs 6 vs 16) and threshold to discriminate absent probe sets (30% vs 100% vs NA). In our analysis, many genes/ESTs differentially expressed in MM BM-MSCs were involved in important processes such as cell cycle, DNA repair, cell adhesion and metabolism. The profiles of biological functions, in which the majority of the genes/ESTs were involved, were highly similar to those observed by Wagner et al. [27] in the replicative senescence of human BM-MSCs. In addition to the reduced proliferative capacity that we and others have observed [16], [18], [32], the expression of SA β-Gal and increase in cell size indicate an early senescent state in MM BM-MSCs *in vitro*. Furthermore, the cell cycle distribution of MM BM-MSCs indicated that the reduced proliferation was not due to increased apoptosis but was related to an accumulation of cells in S phase. Such an accumulation has already been observed in MSC-like cells derived from human gastric cancer [33]. The real-time RT-PCR analyses allowed us to find key factors potentially involved in this S phase arrest. Indeed, S-phase activators (CDK2, Cyclin E and CDC25A) were underexpressed, whereas S-phase inhibitors (p53 and p21) were overexpressed.

According to the emerging consensus [34], the increased expression of p53 and p21 but not p16 and pRB suggested that the cause of senescence was DNA damage. We found, by microarray analyses, that many genes involved in DNA repair were differentially expressed (e.g., TOP2A, BRCA1, RAD51, RFC3, FANCG, DNMT1). Garayoa et al. reported several non-recurrent chromosomal gains and losses in MM BM-MSCs but these results are controversial because the observations of Todoerti et al. did not confirm these DNA alterations [31], [35], [36].

Senescent cells also secrete many biologically active proteins and present a phenotype termed “Senescence-Associated Secretory Phenotype” (SASP) [37] which can be divided into several categories. Among the soluble signaling factor category, we demonstrated that the production of IL-6, IL-1, MCP-1, IGF and OPG was altered in MM BM-MSCs. By cytokine membrane array or microarray, we observed an overexpression of MMP2/9 and uPAR, members of the protease and inhibitor category. Furthermore, the absence of an increased expression of p16 was consistent with the presence of a SASP [37].

Then, we analyzed the osteoblastogenic capacity of MM BM-MSCs because abnormal differentiation is linked to senescence and aging in BM-MSCs [28], [38], [39]. We demonstrated defects in this process characterized by reduction in calcium deposits, which was also observed by Corre et al. in their preliminary results [16]. This reduction could be due to an early overexpression of ALP, but this hypothesis requires additional research. The decreased osteogenic potential of MM BM-MSCs has been reported by different groups and seems associated with increased inflammatory cytokines [18], [40], dysregulated EphB2/EphB4 signaling [41] and canonical Wnt/β-Catenin pathway inhibition [42]. We and others have observed constitutive overexpression of DKK1 (a Wnt inhibitor) in MM BM-MSCs which may explain the aggressive and irreversible destruction of bone in MM patients [43].

The mechanism of pro-hematopoietic function of MSCs is associated with the production of a sustained release of secretory molecules that promote HSC self-renewal and maintenance [44]. Members of the interleukin family (IL-6 and IL-8), and growth factors (SCF, IGF-2 and HGF) are important players in HSC expansion [45]–[47]. Recently, it has been reported that MSCs, derived from umbilical cord blood, provide a better source of stromal support cells for CD34^+^ expansion, linked to their greater production of IL-6, IL-8, SCF, HGF and MCP-1 [48]. In our study, we have observed a higher secretion of many factors such as HGF, IL-6, IL-8, SCF, IGF-2 and MCP-1 by MM BM-MSCs probably in relation with their sustained hematopoietic support.

Lastly, MSCs display immunoregulatory activities and exert their effects on all effectors of the immune response by both direct cell-cell contacts and the production of soluble mediators. We have previously reported that the immunosuppressive potential of MSCs is not constitutive, which implies the presence of a balance between the inhibitory and stimulatory abilities of MSCs [21]. At low MSC/T cell ratios, MSCs display a stimulatory profile and IL-6 is in part responsible for supporting T cell proliferation by preventing apoptosis [49]. At high ratios, that favor and enhance cell-cell interactions, MSCs become suppressive and acquire an inhibitory profile producing immunoregulatory factors such as PGE-2, TGF-β and HGF [50]. In agreement with Arnulf et al. [30], we demonstrated the impaired ability of MM BM-MSCs to inhibit T cell proliferation. However, contrary to Arnulf et al., we performed our experiments with CD3+ purified cells to ensure the specificity inhibition of proliferation. This reduced inhibition could be linked, at least partially, to the alterations in the secretory profile of MM BM-MSCs and notably to the intrinsic decrease in TGF-β production and the increase in IL-6.

Collectively, our observations demonstrated that, in the absence of MM-PCs, MM BM-MSCs became rapidly senescent with profound alterations in their properties. In contrast, the MM-PCs cultured alone in vitro died rapidly unless they were co-cultured with BM-MSCs [51], [52]. We hypothesize that the interactions between MM-PCs and MM BM-MSCs during pathogenesis lead to their co-dependence. To confirm this hypothesis, we need to evaluate if co-cultures of MM BM-MSCs with MM-PCs could restore the normal behaviour of MM BM-MSCs (i.e., proliferation, SA β-Gal expression and p53 expression/activation). Contrary to Zdzisinska et al. [53], our preliminary experiments indicate that the interactions with myeloma plasma cells are necessary to restore the normal proliferation of MM BM-MSCs (data not shown). This opposite result is mainly due to the type of cells used in the co-cultures because Zdzisinska et al. used stromal independent cell line (RPMI 8226) instead of primary myeloma plasma cells in our experiments.

As a possible consequence of this co-dependence, the eradication of MM-PCs by therapy may induce the MM BM-MSCs to enter senescence. In turn, this cellular state could participate in disease relapse by modifying the bone marrow organization and creating a new drug-resistant microenvironment. Furthermore, the disturbance of the bone marrow environment by senescent MM BM-MSCs could be sufficient to induce secondary cancers such as myelodysplasia and leukemia which have a higher risk to develop in MM patients [54]–[56]. Indeed, senescence in stromal cells is linked to the progression of ovarian, skin, breast and pancreatic cancers [57]–[60], and senescent cells could live for prolonged periods. A key factor in this process is the SASP, whose specific members are strong candidates for stimulating malignant phenotypes in neighboring cells [61], [62].

The effects of MM treatments on cancer cells are partially understood but the effects on the surrounding cells are poorly understood, whereas the importance of microenvironment in cancer is increasing. As we demonstrated in this study, current MM treatments are able to reduce MM BM-MSC abnormalities (i.e., secreted factors, proliferation, and osteoblastogenesis). Especially, Bortezomib drastically reduces the DKK1 production, restores a normal production of calcium deposits and a normal profile of ALP activity during MM BM-MSCs osteoblastogenesis. These effects could take part to the beneficial outcomes of Bortezomib-based treatments on myeloma bone disease. It would be very interesting to develop specific treatments to target these abnormalities, or to replace the BM-MSCs, because senescence may be irreversible and most current cancer therapies are toxic and work by increasing oxidative stress and DNA damage [63]. The utilization of MSCs from other tissues (e.g., adipose tissue) would be a good substitute in combination with ASCT. Indeed, the MSC is able to improve engraftment [64] and is a promising candidate for cell-based therapeutic approaches in MM [65].

Finally, it has been hypothesized that MM pathogenesis involves increasing interactions between malignant PCs and their microenvironment, as many of the genetic changes observed in MM are already present in MGUS [66]. In this light, we analyzed some of the characteristics of BM-MSCs from MGUS patients and no differences were observed when compared to HD BM-MSCs. Our preliminary results indicate that the transformation from MGUS to MM requires the involvement of bone marrow microenvironment. However, further analyses are required to understand the evolution of the interactions between the malignant cells and BM-MSCs across all disease stages.

## Supporting Information

Figure S1 **mRNA expression (relative to GAPDH) of ANGPTL1, ANGPTL4, Stat1, Ankyrin 3, CDC25A, CDK2, Cyclin E, FosB and Sox9 by HD BM-MSCs (n = 14), untreated MM BM-MSCs (n = 8) and MM BM-MSCs treated by Lenalidomide (n = 6), Thalidomide (n = 9) and bortezomib (n = 10).** *p<0.05 compared to HD BM-MSCs.
(TIF)

Table S1 **Characteristics of MM patients.**
(DOC)

Table S2 **qRT-PCR Primers.**
(DOCX)
