# Supplementary figures and images for: Evidences of Early Senescence in Multiple Myeloma Bone Marrow Mesenchymal Stromal Cells
Source: PLoS One. 2013 Mar 21;8(3):e59756. doi: 10.1371/journal.pone.0059756 (PMC3605355; doi:10.1371/journal.pone.0059756)

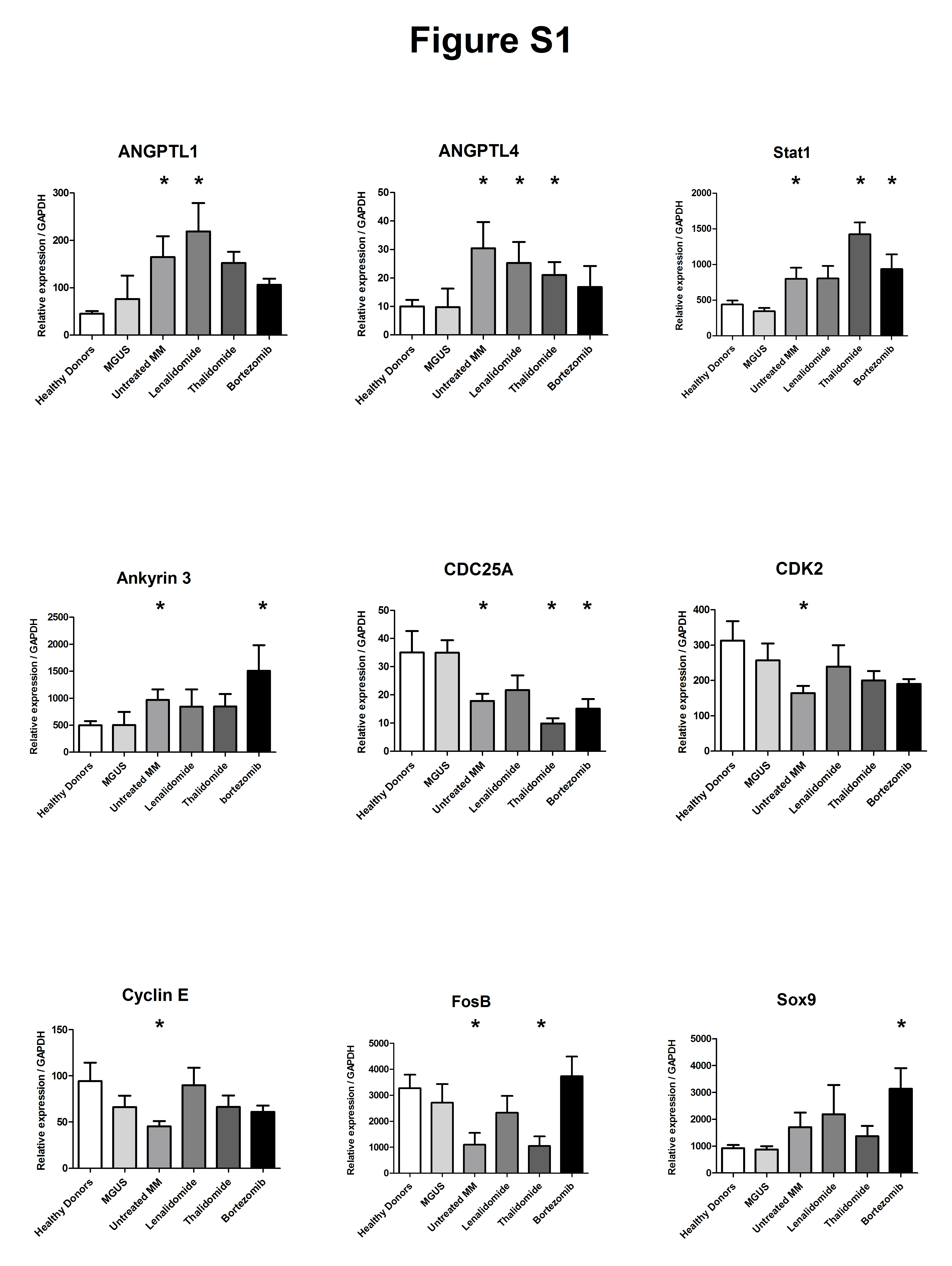

Supplement: Figure S1 — mRNA expression (relative to GAPDH) of ANGPTL1, ANGPTL4, Stat1, Ankyrin 3, CDC25A, CDK2, Cyclin E, FosB and Sox9 by HD BM-MSCs (n = 14), untreated MM BM-MSCs (n = 8) and MM BM-MSCs treated by Lenalidomide (n = 6), Thalidomide (n = 9) and bortezomib (n = 10). *p<0.05 compared to HD BM-MSCs. (TIF) [file pone.0059756.s001.tif]
