# Supplementary material for: Evidences of Early Senescence in Multiple Myeloma Bone Marrow Mesenchymal Stromal Cells
Source: PLoS One. 2013 Mar 21;8(3):e59756. doi: 10.1371/journal.pone.0059756 (PMC3605355; doi:10.1371/journal.pone.0059756)
Supplement: Table S1 — Characteristics of MM patients. (DOC) [file pone.0059756.s002.doc]

| **Patient categories** | **Number** | **Mean age (range)** | **Sexe ratio (M/F)** | **Salmon Durie stage** | **MM type** |
| --- | --- | --- | --- | --- | --- |
| **MGUS** | **9** | **62 (46-79)** | **2/7** | **NA** | **7 IgG**  **1 IgA**  **1 IgM** |
| **Untreated MM** | **13** | **63 (51-78)** | **7/6** | **9 III**  **4 II** | **9 IgG**  **3 IgA**  **1 IgM** |
| **Lenalidomide-based treatment** | **10** | **61 (50-79)** | **5/5** | **7 III**  **3 II** | **7 IgG**  **2 IgA**  **1 IgD** |
| **Thalidomide-based treatment** | **14** | **61 (41-82)** | **7/7** | **10 III**  **4 II** | **10 IgG**  **4 IgA** |
| **Bortezomib-based treatment** | **11** | **59 (51-68)** | **7/4** | **9 III**  **2 II** | **8 IgG**  **3 IgA** |
