# Supplementary material for: Evidences of Early Senescence in Multiple Myeloma Bone Marrow Mesenchymal Stromal Cells
Source: PLoS One. 2013 Mar 21;8(3):e59756. doi: 10.1371/journal.pone.0059756 (PMC3605355; doi:10.1371/journal.pone.0059756)
Supplement: Table S2 — qRT-PCR Primers. (DOCX) [file pone.0059756.s003.docx]

| **Transcripts** | **Forward** | **Reverse** |
| --- | --- | --- |
| **p53** | **AGGCCTTGGAACTCAAGGAT** | **CCCTTTTTGGACTTCAGGTG** |
| **p21** | **CGAAGTCAGTTCCTTGTGGAG** | **CATGGGTTCTGACGGACAT** |
| **p16** | **TGCCTTTTCACTGTGTTGGA** | **TGCTTGTCATGAAGTCGACAG** |
| **pRB** | **TCCTGAGGAGGACCCAGAG** | **AGGTTCTTCTGTTTCTTCAAACTCA** |
| **CDC25A** | **CGTCATGAGAACTACAAACCTTGA** | **TCTGGTCTCTTCAACACTGACC** |
| **CDK2** | **GCTAGCAGACTTTGGACTAGCCAG** | **AGCTCGGTACCACAGGGTCA** |
| **Cyclin E** | **CTTCACAGGGAGACCTTTTAC** | **CATTCAGCCAGGACACAATAG** |
| **Stat1** | **GACTGAGTTGATTTCTGTGTCTGAA** | **ACACCTCGTCAAACTCCTCAG** |
| **ANGPTL1** | **ATGATGTGGCATAATGGTAAACA** | **AAGTGGGCGCAGTTTCCT** |
| **ANGPTL4** | **TTTTGGTGAACTGCAAGATGA** | **GAAGTCCACTGAGCCATCGT** |
| **ANK3** | **ACCAATCACAATGACCATTCC** | **ACTGAGCAGGCGAAGTGC** |
| **SOX9** | **GTACCCGACACTTGCACAAC** | **TCGCTCTCGTTCAGAAGTCTC** |
| **FosB** | **CCGAGAGGAGACGCTCAC** | **CTGCTGCTAGTTTATTTCGTTCC** |
| **GAPDH** | **AATCCCATCACCATCTTCCA** | **TGGACTCCACGACGTACTCA** |
